# Control of atypical PKCι membrane dissociation by tyrosine phosphorylation within a PB1-C1 interdomain interface

**DOI:** 10.1016/j.jbc.2023.104847

**Published:** 2023-05-20

**Authors:** Mathias Cobbaut, Neil Q. McDonald, Peter J. Parker

**Affiliations:** 1 Signalling and Structural Biology Laboratory, The Francis Crick Institute, London, UK; 2 Protein Phosphorylation Laboratory, The Francis Crick Institute, London, UK; 3 Department of Biological Sciences, Institute of Structural and Molecular Biology, Birkbeck College, London, UK; 4 School of Cancer and Pharmaceutical Sciences, King's College London, London, UK

**Keywords:** atypical protein kinase C, membrane recruitment, tyrosine phosphorylation, cell signaling, cell polarity

## Abstract

Atypical PKCs are cell polarity kinases that operate at the plasma membrane where they function within multiple molecular complexes to contribute to the establishment and maintenance of polarity. In contrast to the classical and novel PKCs, atypical PKCs do not respond to diacylglycerol cues to bind the membrane compartment. Until recently, it was not clear how aPKCs are recruited; whether aPKCs can directly interact with membranes or whether they are dependent on other protein interactors to do so. Two recent studies identified the pseudosubstrate region and the C1 domain as direct membrane interaction modules; however, their relative importance and coupling are unknown. We combined molecular modeling and functional assays to show that the regulatory module of aPKCι, comprising the PB1 pseudosubstrate and C1 domains, forms a cooperative and spatially continuous invariant membrane interaction platform. Furthermore, we show the coordinated orientation of membrane-binding elements within the regulatory module requires a key PB1-C1 interfacial β-strand (beta-strand linker). We show this element contains a highly conserved Tyr residue that can be phosphorylated and that negatively regulates the integrity of the regulatory module, leading to membrane release. We thus expose a hitherto unknown regulatory mechanism of aPKCι membrane binding and release during cell polarization.

Protein kinase C (PKC) isozymes are subject to tight regulation of their catalytic activities rendering them responsive to an array of cellular signals in specific compartments consistent with other AGC kinase superfamily members (1, 2). For classical and novel PKC isoforms, changes in lipid signaling that induce formation of diacylglycerol (DAG) drive activation, and therefore, PKC responses to various G-protein–coupled receptors and receptor tyrosine kinases linked to PLC activity (1). The atypical PKCs (iota and zeta in human) on the other hand do not contain DAG-responsive elements in their regulatory amino termini but instead contain a PB1 domain tethered *via* a pseudosubstrate (PS) linker region to a DAG-unresponsive C1 domain (3). The PS motif is thought to repress catalytic activity by competitively blocking access to substrates (4), while the PB1 domain mediates protein–protein interactions with activating/regulatory proteins such as p62 and Par6, which in turn are also modular proteins allowing for multivalent protein interactions (5, 6).

Many aPKC functions are intricately linked with its recruitment to the plasma membrane. Indeed, the polarity complexes that define apical and basolateral membrane identity bind membranes specific to these compartments (7). The manner in which aPKC is recruited into and excluded from these membrane-associated polarity complexes is still not entirely clear. Since there is an interdependency with other proteins such as Par3 and Cdc42, membrane association was long considered to be indirect occurring through association with these and other partner proteins (8, 9, 10, 11). However two recent studies have shown at that aPKC can associate directly with cell membranes through its regulatory module (defined hereafter in aPKCι as aPKCι^RM^) (12, 13). The membrane-binding determinants within the regulatory module have been identified as the PS region, which contains a stretch of positively charged Arg residues, and the C1 domain. It is unclear hitherto what the relative contribution of each region is. For example, in HEK293 cells, MDCK cells and the *Drosophila* follicular epithelium, substitutions in the PS region render the protein unable to interact with the plasma membrane, whereas in *Drosophila* neuroblasts, the C1 domain has been identified as the dominant membrane interaction module (12, 13).

Here, we approach the question of how multiple aPKC membrane-binding determinants coordinate and how they are coupled. We use Alphafold *in silico* predicted models for the aPKC regulatory module combined with biochemical and cellular assays to validate a continuous surface harboring known membrane-interacting determinants. We show that the PB1 and C1 domains are coupled and oriented though interfacial contacts also aligning the PS motif and C1 domain for membrane interaction. We identify a highly conserved phospho-Tyr residue within the PB1–C1 interface and show that phosphorylation of this residue modulates membrane-binding capabilities of the regulatory module. We propose that this phosphorylation influences the ability of aPKC to associate with and disassociate from membranes in a regulated manner.

## Results

### A continuous membrane-binding surface is oriented within a compact PB1-PS-C1 aPKCι regulatory module

**Figure 1:**
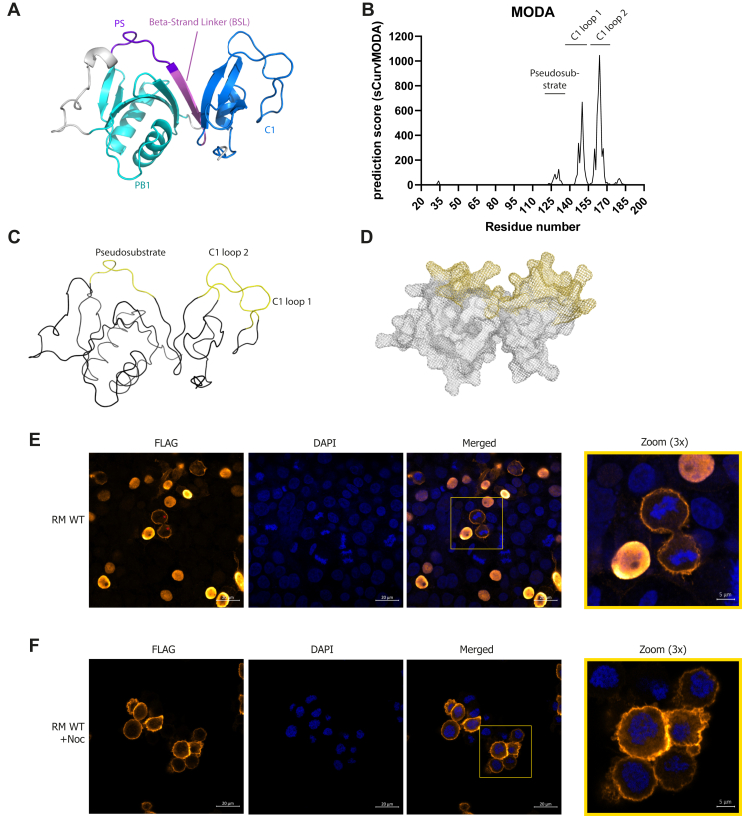
**The aPKCι regulatory module contains a continuous membrane-binding surface and is localized to mitotic cell membranes.***A*, representation of the aPKCι regulatory module as predicted by Alphafold Colab (best scoring model). The individual domains and regions are indicated and *colored*; from N-C PB1 domain (*cyan*), pseudosubstrate (PS) region (*dark purple*), beta-strand linker (BSL) (*light purple*), C1 domain (*blue*). *B*, per residue prediction score of the MODA prediction with above-threshold scoring regions indicated. *C*, cartoon diagram (omitting secondary structure features) of predicted membrane interacting regions from the MODA prediction displayed in the AF Colab model. *D*, mesh representation of panel C showing a continuous sidechain surface. *E*, localization of FLAG-tagged aPKCι regulatory module (RM) in HEK293T cells; *red asterisks* indicate mitotic cells. *F*, localization of FLAG-tagged aPKCι RM in nocodazole-treated (500 nM, 16 h) HEK293T cells. MODA, Membrane Optimal Docking Algorithm.

In light of the recent discrepancies in the relative contributions of PS and C1 domain to aPKC membrane interaction, we took a holistic approach to understand whether the regulatory module acts as a functional unit contributing two coupled membrane-binding determinants. We used AlphaFold Colab to predict the aPKCι^RM^ spanning the PB1 domain, the PS motif, and the C1 domain (14). The top five structural models predict a compact, globular shape with buried interdomain contacts suggesting a coupled functional unit (Fig. S1*A*). The models predict that the PB1 and C1 domains are crucially bridged together *via* an interdomain β-strand which we term beta-strand linker (BSL) (Fig. 1*A*), that is part of a continuous sheet between the β-strands in the PB1 and C1 domains with a parallel orientation to the PB1 and antiparallel to the C1 domain. All of the models show very low predicted alignment errors, indicative of high confidence in the continuous fold (Fig. S1*B*). Using the highest scoring AF Colab model as input, we set out to predict membrane binding residues *via* the Membrane Optimal Docking Algorithm (MODA) package (15). Both the PS and two regions in the C1 domain (termed loop 1 residues 144–153 and loop 2 residues 160–167) have high membrane-association prediction scores (Fig. 1, *B* and *C*; Table S1). Interestingly, both regions form a continuous surface containing both positively charged and hydrophobic residues (Fig. 1, *C* and *D*), potentially contributing to a single composite membrane interaction site. The importance of these residues is further underscored by the fact that most residues constituting the platform are highly conserved as shown *via* ConSurf analysis (Fig. S1*E* and Table S2), while there is less stringency for amino acid composition on the opposite side of the domain (Fig. S1*F*).

**Figure 2:**
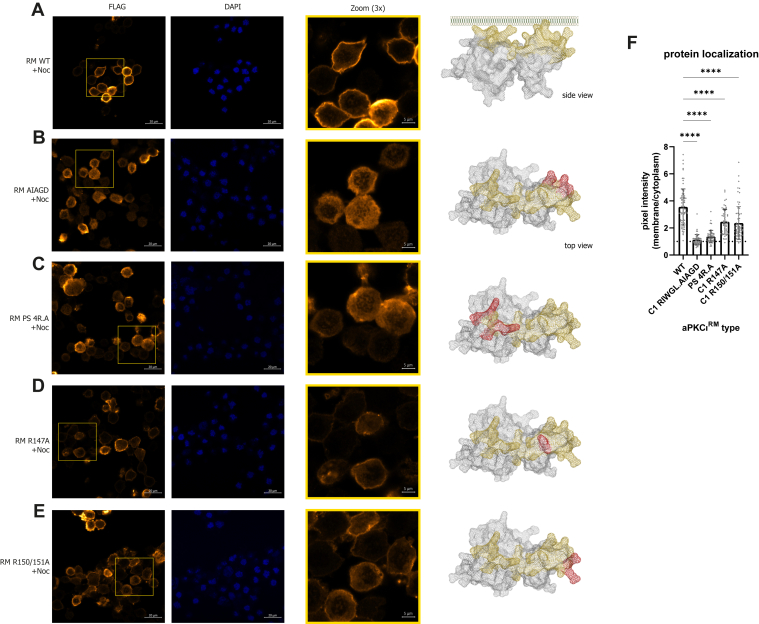
**Probing mutations within the aPKCι^RM^ on membrane binding in mitotic cells reveal a role for both the PS and C1 domain.***A*, localization of WT FLAG-tagged regulatory module (RM) in nocodazole-treated (500 nM, 16 h) HEK293T cells. *B*–*E*, expression of the indicated mutant FLAG-tagged regulatory modules in nocodazole-treated (500 nM, 16 h) HEK293T cells. Mutated residues are depicted on the protein model in *red*. *F*, quantification of membrane/cytoplasmic pixel intensities measured in >45 cells imaged *via* confocal microscopy. Representative experiment of three biological replicates. Differences analyzed *via* one-way ANOVA (∗∗∗∗*p* ≤ 0.0001).

To probe the intrinsic capabilities of aPKCι^RM^ in membrane binding, we expressed FLAG-tagged aPKCι^RM^ in HEK293T cells. We observed a striking localization pattern that was cell cycle dependent. For interphase cells, the regulatory module is predominantly localized in the nucleus (Fig. 1*E*), whereas in mitotic cells, aPKCι^RM^ is uniformly associated with the plasma membrane (Fig. 1*E* asterisks and magnified panel). This observation is in line with those from *Drosophila* neuroblasts where overexpressed aPKCι^RM^ constructs display similar behavior in interphase and at mitosis (13). This property allowed us to assess the membrane-binding requirements of aPKCι^RM^ in a physiological context by arresting cells in mitosis with nocodazole (Figs. 1*F* and S1*G*). To exploit this “mitotic trap” assay, we introduced site-specific mutations targeting residues with the highest relative prediction scores for membrane interaction (Table S1). While the WT regulatory module has a strong (∼4-fold) enrichment at the plasma membrane in nocodazole-treated cells (Fig. 2*A*), we observed a complete loss of membrane binding when substituting the highly predictive ^160^RIWGL^164^ sequence in the C1 domain (loop 2) with ^160^AIAGD^164^ (Fig. 2, *B* and *F*). Similarly, a complete loss of membrane binding was observed with ^126^RRGARR^131^ to ^126^AAGAAA^131^ substitutions in the PS region (termed PS 4R.A, Fig. 2, *C* and *F*). Substitution of Arg-147 or Arg-150/151 to Ala in the C1 domain (loop 1) had a smaller impact reducing membrane binding by about 1.5-fold (Fig. 2, *D*–*F*). This indicates that both regions in the C1 domain and the PS motif contribute to allow optimal membrane binding in these mitotic cells. Based upon the model, it is likely these motifs are organized in a continuous surface, the integrity of which likely dictates the capacity to bind membranes.

### Tyr-136 phosphorylation within an interfacial motif uncouples membrane-binding determinants but does not strongly impact catalytic activity

**Figure 3:**
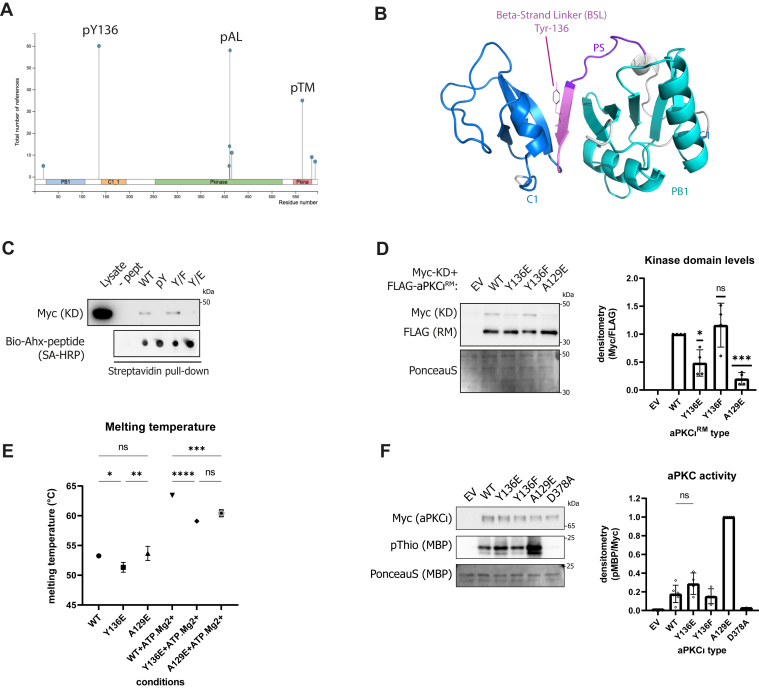
**Tyrosine-136 phosphorylation within the BSL affects aPKCι stability with a minor impact on catalytic activity.***A*, identification of main phosphorylation sites on aPKCι identified in the Phosphosite database. *B*, visualization of Tyr-136 central to the BSL region of the regulatory module, color coded as in Fig. 1*A*. *C*, peptide-pulldown of Myc-tagged aPKCι kinase domain with extended pseudosubstrate peptides (Biotin-Ahx-SIYRRGARRWRKLYCANGHT-CONH2) containing the indicated modifications at Y^14^, *i.e.*, pY, Y/E and Y/F. *D*, co-expression of FLAG-tagged regulatory module and Myc-tagged kinase domain of aPKC in HEK293T cells. Equal amounts of DNA were transfected and stabilization of Myc-tagged kinase domain by different mutants of the regulatory module was followed. Quantification of Myc-kinase domain levels relative to WT aPKCι^RM^ co-expression shown in the *right panel* (n = 4 biological replicates, average ± SD, analyzed *via* one-sample *t* test (v.a.v. a value of 1): ns *p* > 0.05, ∗*p* ≤ 0.05, ∗∗∗*p* ≤ 0.001). *E*, DSF assay measuring thermal stability of WT and mutant fl-aPKCι in absence or presence of ATP.Mg^2+^. Plotted is the T_m_ derived from the inflection point of the melting curve (using the derivative maximum) (average ± SD, analyzed *via* one-way ANOVA: ns *p* > 0.05, ∗*p* ≤ 0.05, ∗∗*p* ≤ 0.01, ∗∗∗*p* ≤ 0.001, ∗∗∗∗*p* ≤ 0.0001). *F*, *in vitro* kinase assay with immunoprecipitated WT or mutant kinases on MBP. Incorporation of new phosphates into MBP was followed with ATP-γS. Quantification of MBP thiophosphate levels shown on the right (n = 4 biological replicates, average ± SD, analyzed *via* unpaired *t* test: ns *p* > 0.05). MBP, myelin basic protein; PKC, protein kinase C.

One of the more salient features of the regulatory module is the interfacial BSL motif positioning the PB1 and C1 domain, orienting the membrane-binding determinants within the aPKCι^RM^. The BSL shows overall high conservation centered on a centrally positioned, highly conserved Tyr residue 136 (Fig. S1*H* and Table S2). Inspection of the human PRKCI entry in Phosphosite (https://www.phosphosite.org/) indicated that this residue is frequently phosphorylated in more than 60 high-throughput studies in a variety of conditions and cell types, alongside the familiar activation loop (residue T412) and turn motif (residue T564) priming sites (Fig. 3*A*) (16). Consistent with this, it is also one of few aPKCι phosphoresidues identified in the MaxQuant Database (MaxQB) (Table S3) (17).

As Tyr-136 lies at the core of the regulatory module within the interfacial BSL motif and proximal to the PS (Fig. 3*B*), we considered whether tyrosine phosphorylation could influence the full-length kinase conformation, stability, and activation state. First, we probed whether phosphorylation affects the interaction between the PS region and the kinase domain. By definition, the PS is proposed to bind the catalytic domain at the substrate-binding pocket to be released upon aPKCι activation. We wondered whether in a linearized context, Tyr phosphorylation proximal to the PS could influence this behavior. To test this, we immunoprecipitated a Myc-tagged kinase domain (aPKCι^KD^) from cell lysates using a biotin-labeled extended PS peptide containing either a nonphosphorylated Tyr or pTyr residue or Glu or Phe substitutions to mimic either phosphorylated or nonphosphorylated states, respectively (Fig. 3*C*). We saw specific binding of the peptide to the core kinase domain and observed loss of kinase domain interaction with the peptide when the Tyr residue is either phosphorylated or substituted with Glu. Conversely, a nonphosphorylated or Phe-substituted peptide effectively captured the aPKCι^KD^. To confirm whether the phosphorylation of this residue reduces the interaction between aPKCι kinase domain and its regulatory module, we assessed the protection of the kinase core by co-expressing the isolated kinase domain with either wildtype (WT) or mutant forms of aPKCι^RM^ (Fig. 3*D*). While the WT aPKCι^RM^ protein product protects the aPKCι^KD^, a Y136E substitution causes a reduction in the levels of aPKCι^KD^ protein, as does a previously described PS disengaged A129E mutant (4). These results indicate that the kinase domain-regulatory module interaction is weakened by phosphorylation of Tyr-136.

To probe whether this effect is due to loss of direct binding of the PS region to the kinase domain or the aPKCι^RM^ conformation, we purified WT, Y136E, and A129E mutant full-length aPKCι (aPKCι^FL^) from HEK293FS cells and subjected them to differential scanning fluorometry (Fig. 3*E*). In the apo-form, the melting temperature (T_m_) for the WT protein is 53.3 °C, similar to the A129E mutant (T_m_ = 53.7 °C). The Y136E mutant is less thermostable with a T_m_ of 51.3 °C in the apo-form. Addition of ATP.Mg^2+^ stabilizes both WT and mutant kinases; however, comparatively, the A129E mutant is stabilized to a lesser degree than WT (A129E ΔT_m_ = 6.7 °C *versus* WT ΔT_m_ = 10.2 °C). This suggests that occupation of the active site with ATP, while stabilizing the kinase core, may result in a less thermostable open conformation, possibly because of phosphate-driven charge repulsion with the Glu substitution. The Y136E mutant is less thermostable than WT in both the presence and absence of ATP, indicating that decreased thermostability is caused by mutation-driven alterations in the regulatory module.

To test the impact of Tyr-136 phosphorylation on aPKCι catalytic activity, we followed the phosphorylation of myelin basic protein (MBP) *in vitro* after immunoprecipitation of WT and mutant forms of aPKCι^FL^. To monitor the incorporation of new phosphate groups on MBP, we made use of γ-thiophosphate ATP (Fig. 3*F*). MBP was readily phosphorylated by the conformationally “open” A126E mutant but showed only a marginal nonsignificant increase in phosphorylation upon incubation with a Y136E mutant compared to WT. This indicates that in the ‘tethered’ state of the full-length protein, the impact of the weakened regulatory module/catalytic domain interaction consequent to Tyr-136 phosphorylation is modest compared to the PS A129E mutation, while there is a strong effect on the overall aPKCι^RM^ conformation.

### Tyr-136 phosphorylation disrupts aPKCι^RM^ membrane binding in cells

**Figure 4:**
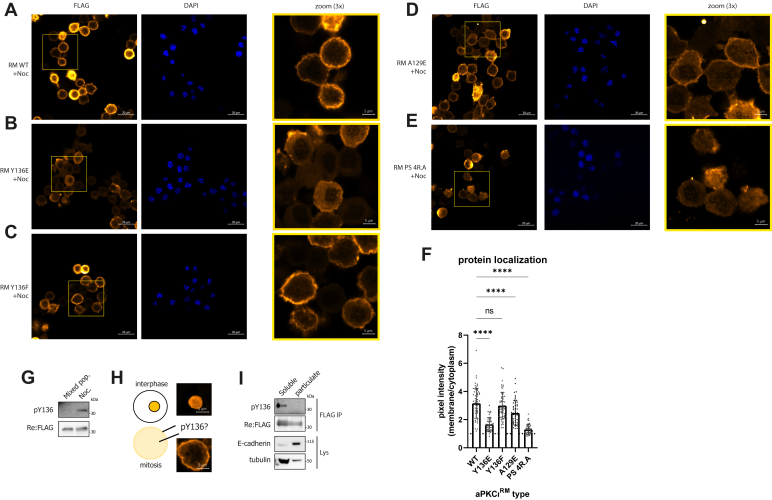
**Tyrosine-136 phosphorylation within the BSL correlates with cytoplasmic aPKCι^RM^.***A*–*E*, expression of the indicated mutant FLAG-tagged regulatory modules (RM) in nocodazole-treated (500 nM, 16 h) HEK293T cells. *F*, quantification of membrane/cytoplasmic pixel intensities measured in >45 cells imaged via confocal microscopy. Representative experiment of three biological replicates. Differences analyzed *via* one-way ANOVA (ns *p* > 0.05, ∗∗∗∗*p* ≤ 0.0001). *G*, Western blot analysis of phospho-Tyr-136 levels in the in untreated (mixed pop.) cells or cells treated with 500 nM nocodazole for 16 h (Noc.). *H*, representation of the observed localization of regulatory module species and possible mapping of its phospho-form. *I*, fractionation of cells by ultracentrifugation and probing for Tyr-136 phosphorylation of the regulatory module (representative experiment of n = 2 biological replicates).

Since the above results indicate a change in regulatory module conformation with little impact on activity, we tested whether Tyr-136 phosphorylation had a direct influence on the aPKCι^RM^ membrane binding properties. We assessed membrane association in mitotic cells as above, expressing the WT regulatory module (Fig. 4*A*) or mutants in nocodazole-treated cells; the PS 4R.A mutant was used as a control for loss of membrane binding. Substitution of Tyr-136 with Glu resulted in a significant 1.5-fold reduction of membrane intensity (Fig. 4, *B* and *F*), whereas a Tyr-Phe substitution had a negligible impact on membrane binding (Fig. 4, *C* and *F*), consistent with Tyr phosphorylation being disruptive for membrane interaction. Interestingly, a substitution of A129 to Glu had only a small but significant impact on membrane binding capabilities, despite its negative charge central to the PS region (Fig. 4, *D* and *F*), whereas substitutions of the PS Arg residues resulted in a complete loss of membrane binding (Fig. 4, *E* and *F*).

To probe Tyr-136 phosphorylation in the regulatory module of aPKC, we developed a site-specific and phospho-specific rabbit antibody. Initial attempts at immunizing rabbits with the peptide surrounding Tyr-136 resulted in poor and nonspecific immunoreactivity of the serum, but using a derivatized phosphopeptide with S-methylcarboxyamide coupled to the adjacent Cys residue C-terminal to the phosphorylated Tyr residue as the immunogenic peptide, we were able to detect Tyr phosphorylated Myc-aPKC in pervanadate-treated cells after immunoprecipitation using purified phosphoantibodies (Fig. S2, *A* and *B*). Alkylation of nitrocellulose membranes with iodoacetamide greatly improved epitope recognition, confirming recognition of the double-modified epitope (Fig. S2*A*). Interestingly, using this antibody, we could show significant levels of Tyr-136 phosphorylation in regulatory module constructs in nocodazole-treated cells (Figs. 4*G* and S2*C*). This led us to ask the question whether the phosphorylated species segregates to the membrane or cytosolic compartment in mitotic cells (Fig. 4*H*). To assess any preferential localization of the phosphorylated species, we fractionated nocodazole-treated cells by ultracentrifugation (100,000*g*) to separate membrane (particulate) and cytosolic (soluble) components. We could detect no phosphorylation of the regulatory module in the particulate fraction indicating that this species does not associate with the membrane fraction, while phosphorylated protein was detected in the cytosol (Fig. 4*I*). This further supports our observation with the Glu phosphomimetic mutant, showing that tyrosine phosphorylation impacts the protein cycling between membrane and cytosolic fractions.

**Figure 5:**
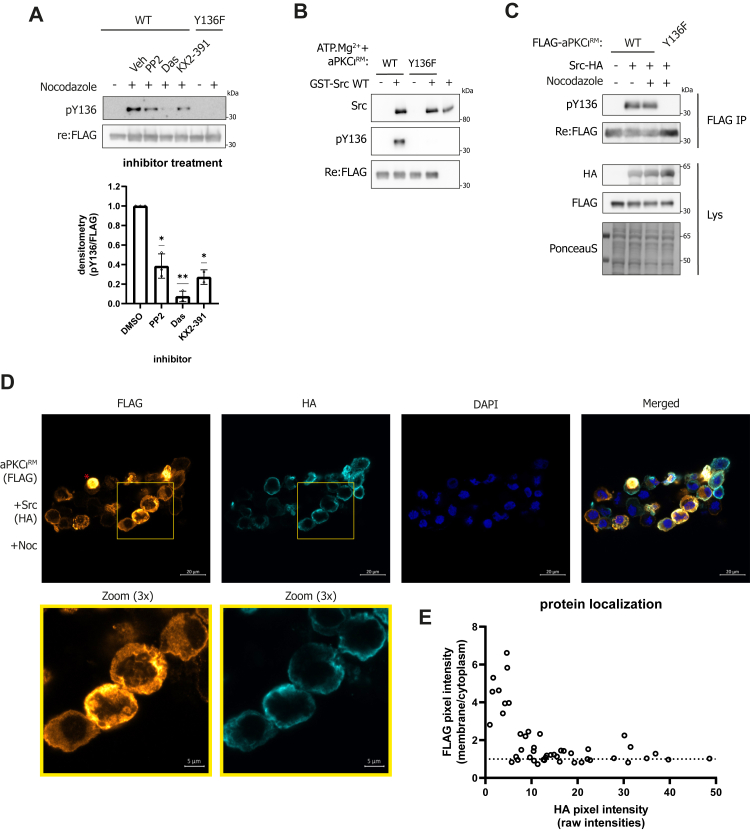
**Src phosphorylates Tyr-136 and influences aPKCι^RM^ localization.***A*, effect of Src inhibition on Tyr-136 phosphorylation. HEK293T cells expressing FLAG-tagged regulatory module were treated with nocodazole (500 nM, 16 h) or *left* untreated and incubated with the indicated inhibitors (PP2 10 μM, dasatinib 1 μM or KX2-931 1 μM) for 1 h before lysis and probed for Tyr-136 phosphorylation. Quantification of pY136 levels shown in the right panel (n = 3, average ± SD) analyzed *via* one-sample *t* test (v.a.v. a value of 1; ∗*p* ≤ 0.05, ∗∗*p* ≤ 0.01); *B*, *in vitro* phosphorylation of the aPKCι^RM^ with Src. WT or Y136 F aPKCι^RM^ was immunoprecipitated from HEK293T cells and incubated with Src (20 ng) and ATP.Mg^2+^ for 20 min after which the reactions were detected for Tyr-136 phosphorylation. *C*, levels of Tyr-136 phosphorylation upon co-expression of aPKCι^RM^ with Src-HA in HEK293T cells treated with nocodazole or left untreated. *D*, localization of aPKCι^RM^ upon co-expression with Src-HA in nocodazole (500 nM, 16 h) treated HEK293T cells. Mitotic cells with high levels of Src-HA display increased cytoplasmic staining for aPKCι^RM^ compared to cells that only express aPKCι^RM^. (The *red asterisk* indicates an interphase cell where aPKCι^RM^ is nuclear). *E*, quantification of membrane/cytoplasmic FLAG pixel intensities plotted against the HA pixel intensities in 46 cells imaged *via* confocal microscopy. PKC, protein kinase C; RM, regulatory modules.

In addressing the regulation of this Tyr-136 phosphorylation, we considered the possibility of autophosphorylation. The Y136 phosphorylation of the ‘free’ aPKCι^RM^ in mitotic cells, suggested that this was not due to the aPKCι kinase domain exhibiting Tyr specificity due to proximity in *cis* (*in casu* the PS motif), as seen in some other Ser/Thr kinases as recently observed in the PKDs (18). Indeed, we could detect no phosphorylation of Tyr-136 upon incubation of aPKCι^FL^ with ATP.Mg^2+^ (data not shown). Exploring candidate upstream kinases, we pulled down proteins from a cell extract using a biotinylated extended PS peptide containing either a Y, F, or pY residue corresponding to Tyr-136 and then spiked the precipitated protein mixture with ATP.Mg^2+^, allowing any associated kinase to (auto)phosphorylate. Western blot analysis with a phospho-Tyr antibody showed tyrosine kinase (autophosphorylation) activity around ∼60 kDa and ∼170 kDa in protein mixtures associating with the Y and F but not the product pY peptide (Fig. S3*A*). *In silico* predictions of potential upstream kinases using Netphos3.1 (19) and NetPhorest (20) score Src among the top predicted kinases, which could correspond to the autophosphorylation activity observed at ∼60 kDa (Fig. S3*B*). Although the sequence surrounding Tyr-136 is basic, the major determinant for Src selectivity for a hydrophobic residue at the −1 position is satisfied (Fig. S3*C*). In a subsequent screen with a panel of Tyr kinase inhibitors, we found responsiveness of aPKCι^RM^ Tyr-136 phosphorylation in nocodazole-treated conditions to the Src family kinase (SFK) inhibitors PP2 and dasatinib, as well as the Src-specific inhibitor KX2-391 which binds to the Src peptide binding cleft (Fig. 5*A*) (21). To test direct Src phosphorylation of Tyr-136, we incubated the aPKCι^RM^ with Src *in vitro.* This resulted in significant levels of Tyr-136 phosphorylation (Fig. 5*B*). Additionally, co-expression of Src and the aPKCι^RM^ results in high levels of Tyr-136 phosphorylation in both interphase and mitotic HEK293T cells (Fig. 5*C*) as well as an increased cytoplasmic pool of aPKCι^RM^ in cells with high Src expression (Fig. 5, *D* and *E*). These data support Src acting as an upstream kinase for Tyr-136, influencing aPKCι^RM^ localization.

**Figure 6:**
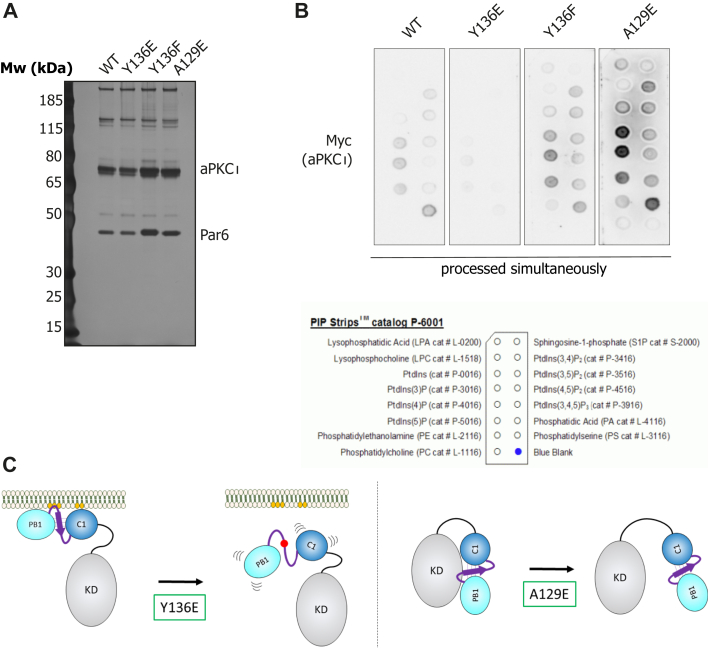
**Lipid**-**binding preferences of aPKCι mutants and model for the effects of Tyr-136 phosphorylation compared to PS disengagement.***A*, silver-stained SDS-PAGE gel of the Myc-aPKC-2xStrep-Par6 binary complexes purified through 2xStrep-Par6. *B*, lipid overlay assay using the protein complexes defined in (*A*). Levels of aPKCι bound to the PIP-strip membrane were quantified using anti-Myc tag antibody. *C*, schematic representation of the impact of mutants and phospho-states of aPKCι. The *left panel* displays the anticipated consequence of Tyr-136 phosphorylation in the BSL as mimicked by a Y136E substitution, disrupting the continuous membrane binding platform constituted by the PS and C1 domain. The right panel displays the impact of a pseudo-substrate disengaged conformation, mimicked by an A129E substitution which mainly results in relieved auto-inhibition and increased catalysis. BSL, beta-strand linker; PS, pseudosubstrate; PKC, protein kinase C.

To further assess direct lipid binding in the context of the full-length protein, including the effects of increased dynamics between regulatory and kinase domain in the mutants, we purified the aPKC–Par6 complex from HEK293T cells using Par6 as bait (Fig. 6*A*). The binary complex is thought to be in a more open conformation as Par6 is proposed to displace the PS region, potentially exposing the aPKCι^RM^ for lipid binding (4). These binary protein complexes were then used in a lipid overlay assay with PIP-strips (Fig. 6*B*). The WT complex interacts preferentially with monophosphorylated inositol headgroups, (PI3P, PI4P, and PI5P) but also with doubly phosphorylated headgroups (PI(3,4)P_2_, PI(3,5)P_2_, and PI(4,5)P_2_) with no strong preference for the position of the phosphate (Fig. 6*B*). The complex also binds phosphatidylserine while we observed no binding to sphingosine-1-phosphate which was previously described as a lipid activator for aPKCs (22). Substituting Tyr-136 with Glu results in lower immunodetection when processed in parallel, indicative of lower apparent binding to all phospholipids, whereas a Phe mutant displays WT levels of detection (Fig. 6*B*). Interestingly, a PS-disengaged mutant (A129E) displays increased immunodetection when incubated with PIP-strips despite the glutamic substitution. This is indicative of the fact that this mutation can further drive the Par6-bound open conformation, allowing for enhanced lipid binding.

## Discussion

Cell polarity—the spatially asymmetric organization of a cell and its components—is an essential biological process, orchestrated by key regulatory inputs. One central component of this process conserved in higher eukaryotes is atypical protein kinase C. Together with its partner Par6, this kinase is involved in regulating a variety of protein complexes, prominently those include Par3, Lgl1/2, Crb, but also Dlg, Par1, FARP2, and others to polarize epithelial cells alongside an apico-basal axis (23, 24, 25, 26, 27, 28, 29). While there are functional data on the interdependency of polarity components, molecular insight into the regulation of these assemblies and their distribution is still lacking. For example, it is not clear how the aPKCs are recruited to membranes and membrane-associated protein assemblies. Several studies have shown a dependency on Par3 (8, 9, 10, 11); however, Par3-independent mechanisms have also been described (30). The correct lipid environment plays a crucial role in recruiting and tethering the right polarity components, with anionic phospholipids and prominently PI(4,5)P_2_ playing a dominant role in the apical domain (31, 32). Several polarity proteins respond to these lipids *via* basic-hydrophobic motifs (33, 34). Interestingly, in a recent study, the PS of aPKC was identified as such a lipid-responsive basic-hydrophobic domain (12). This aligns with the long-standing notion that the PKC PS region interacts with negatively charged lipids (35). In addition, the response to PIP3 downstream of insulin has also been mapped previously to the PS region (36). However, a subsequent study by the Prehoda group showed that in dividing *Drosophila* neuroblasts membrane targeting was mainly dependent on the C1 domain also *via* a phospholipid-binding mechanism (13).

Here, we have investigated membrane binding by aPKCι^RM^ making use of recent advances in AI by Alphafold to derive a structural model for multidomain modules of proteins. While attempts at purification of aPKCι^RM^ for structure determination were unsuccessful, AlphaFold Colab predictions indicated it adopts a single compact unit comprising the PB1 and C1 domains, in which they are tethered *via* an interdomain β-strand proximal to the PS embedded in a continuous sheet spanning across both domains. Based on this predicted model, we used the MODA prediction algorithm which identified three regions containing putative membrane-binding residues. Interestingly, together these predicted membrane-binding regions form a highly conserved continuous platform in the aPKCι^RM^ model. Aside from the PS sequence ^126^RRGARRWRK^134^, we identify ^160^RIWGLGRQ^167^ in the C1 domain as a second sequence strongly contributing to membrane binding. Mutagenesis in either of these sequences completely disrupts membrane binding in the mitotic cell assay, indicating that neither alone provides sufficient affinity, and an intact platform is required to establish the avidity effect for optimal membrane binding in these cells. Additionally, Arg-147 and Arg-150/151 in the C1 domain also contribute albeit to a lesser degree.

The organization of the membrane binding platform is crucially driven by the interfacial BSL motif, with at its core an evolutionarily conserved phospho-Tyr residue that allows for regulatory input. Interestingly, we observed this dynamic behavior in mitotic cells where we found that aPKCι Tyr-136 was phosphorylated only in the cytoplasm. Whether the released protein retains an active, open conformation impacting cytosolic targets is yet to be determined, although our evidence indicates that a tyrosine phosphorylation mimetic alone is not substantially activating despite weakening the inhibitory interaction of the regulatory module on the catalytic domain (Table 1). Based on the effects on thermostability, we propose that the PB1 and C1 domains become discontinuous when Tyr-136 in the BSL is phosphorylated, compromising the overall conformation and membrane binding in mitotic HEK cells (Table 1 and Fig. 6*C*, left hand side). This distinguishes the BSL from the PS, which besides being part of the aPKC membrane interaction module, regulates kinase activity by direct interaction with the kinase domain evidenced by a loss of kinase domain stabilization and increased catalytic activity upon A129E substitution (Table 1). The PS is therefore the main regulatory unit influencing catalysis (Fig. 6*C*, right hand side).

**Table 1:** Effects measured in the respective assays described in this study

Color-coded based on the average measurements obtained in the assays, individual cells are colored from red (low) to yellow (intermediate) to green (high).

Tyr phosphorylation in PKC regulatory domains is not restricted to aPKCs. Interestingly, in PKCδ Tyr-155 phosphorylation in the inter PS-C1a region has also been identified. While reduced nuclear uptake and apoptosis and increased proliferation have been attributed to nonphosphorylatable mutants, conditions in which this Tyr is phosphorylated and how this may impact on membrane binding and catalytic activity have not been well studied (37, 38, 39, 40).

The observation that in aPKCι, there is a pTyr residue central to the regulation of membrane binding implicates cross-talk with Tyr kinase activities contributing to a polarized distribution of the aPKCs. The role for Tyr kinase activity in junction formation and cell polarity was clear early on (41, 42, 43); however, few molecular links have been established. We show for Tyr-136 that SFKs are potential upstream regulators. While we can positively confirm that Src can phosphorylate aPKCι^RM^ *in vitro,* and on co-expression, we should note that the inhibitors PP2 and KX2-391 did not fully abolish Tyr-136 phosphorylation and that dasatinib and PP2 are furthermore not Src specific, implying the possibility of some redundancy. Interestingly, Src has long been known to associate with aPKCs, docking to their PXXP-rich motifs N-terminal to the PS site; an *in vitro* phosphorylation reaction with Src using aPKC-derived peptides also included a peptide containing pTyr-136, further supporting the fact that SFKs can act upstream (44, 45). The cell-cycle dependency of Tyr-136 phosphorylation we observed raises intriguing questions for further exploration regarding the Src-aPKC connection in asymmetrical cell division. v-Src transformation of the zebrafish embryo enveloping layer epithelium causes mitotic-dependent extrusion, partially by affecting cell polarity through Cdc42 and aPKC (46). Src also promotes epithelial polarity switching in colorectal cancer spheroids, promoting a switch from apical-in (central lumen) to apical-out oriented metastatic cell clusters (47). Other Src family kinases such as Fyn and Lyn have also been involved in the regulation of cell polarity, opening up further possibilities of connecting PTK and polarity pathways (48, 49).

Recruitment and retainment of aPKC at membranes involves a variety of factors that are either PKC intrinsic or extrinsic through membrane-anchored proteins. This complex mesh allows for a variety of inputs and affinity thresholds. We show here the mechanism by which aPKCs can interact with membranes through a continuous interaction platform spanning the whole regulatory region, orchestrated by an interdomain β-strand that can be regulated *via* tyrosine phosphorylation. The hierarchy and local mechanisms by which aPKCs are recruited and retained at the correct membrane locus, as well as a further understanding of tyrosine kinase crosstalk with cell polarity, are intriguing questions for future exploration.

## Experimental procedures

### In silico/molecular modeling

To predict the tertiary structure of the regulatory domain, we used Alphafold Colab (https://colab.research.google.com/github/sokrypton/ColabFold/blob/main/AlphaFold2.ipynb) (14) with residues 22 to 194 of human aPKCι as input. Amber forcefield was on, MSA mode was MMseqs2, pair mode was unpaired+paired, model type was auto, and the number of recycles was 12. The top scoring model coordinates were used for subsequent prediction of membrane binding motifs using the MODA (15) and ConSurf (50).

### Cell lines and reagents

HEK293T cells were grown in Dulbecco’s modified eagle medium supplemented with 10% (v/v) fetal bovine serum (ThermoFisher Scientific), 100 U/ml penicillin, and 100 μg/ml streptomycin (ThermoFisher Scientific) at 37 °C in 5% CO_2_. HEK293FS cells were grown in Freestyle 293 Expression Medium (ThermoFisher Scientific) in a shaking incubator at 37 °C in 8% CO_2_. Cells were obtained from the institute’s central catalog, where they are routinely checked for *mycoplasma*. Anti-thiophosphate ester antibody was from Abcam. Anti-FLAG M2 antibody and FLAG-agarose resins were purchased from Sigma. Strep-Tactin II agarose was from GE HealthCare. Myc-trap agarose was from Chromotek. Anti-Myc, secondary HRP-linked goat anti-Rabbit, and Horse anti-Mouse antibodies were from Cell Signaling Technologies. GST-tagged Src was from Carna Biosciences. Polyethyleneimine (PEI) was from Polysciences Inc. Mutagenesis and cloning were done using In-Fusion (Takara). Primers and plasmids used are listed in Table S4. Peptides used were made in-house.

### Protein expression and purification

For expression of Strep-tagged Par6 co-expressed with Myc-tagged fl-aPKCι adherent HEK293T cells were transiently transfected using PEI at a 1:3 (m/m) plasmid/PEI ratio. Forty-eight hours post-transfection, cells (4 10 cm dishes at confluency, ∼4 × 10^7^ cells) were lysed in 50 mM Tris, pH 7.4, 150 mM NaCl, 1% Triton, 0.5 mM TCEP supplemented with phosphatase inhibitors (PhosSTOP, Roche), and protease inhibitors (cOmplete, Roche). Cell lysates were incubated with Strep-Tactin II (GE HealthCare) affinity beads for 2 h at 4 °C while rotating. Next, the beads were washed twice with lysis buffer supplemented with NaCl to 500 mM, once with lysis buffer, and once with elution buffer (50 mM Tris pH7.4, 150 mM NaCl, 0.5 mM TCEP). The protein was eluted in elution buffer supplemented with 2.5 mM D-desthiobiotin. For the purification of 2xStrep-tagged fl-aPKCι, the same protocol was followed, but HEK293FS cells (∼2 × 10^8^ cells) were used and transfected for 96 h instead of 48 h. In addition, a size-exclusion chromatography step was performed on a Superdex-200 increase column, and peak fractions were pooled, dialyzed against elution buffer, and concentrated. Protein quantity and purity was assessed using SDS-PAGE and A280.

### Antibody production

The phospho-Tyr-136 antibody used in this study was made using an immunogenic peptide spanning residues 125 to 141 of human aPKCι synthesized with modifications at Tyr-136 (phosphate) and Cys-137 (S-methylcarboxyamide) and included an additional N-terminal Cys for coupling reactions. New Zealand White rabbits were injected (off-site on commission by White Antibodies) with KLH coupled antigen (500 μg) and Freund’s complete adjuvant and subsequently on day 14, 28, 42, 56, and 70 with antigen and Freund’s incomplete adjuvant. The serum was collected on day 77, and reactive antibodies were purified from serum by sequential purification on a nonphosphopeptide column (flowthrough) and a phospho-peptide column (eluate). These were prepared by binding peptides to SulfoLink resin according to the protocol of the manufacturer (Thermo Fisher Scientific). Antibodies were eluted from the phospho-peptide column using 100 mM glycine pH 3.0 and immediately neutralized with 1 M Tris-HCl, pH 8.

### *In vitro* kinase assay

HEK293T cells (10 cm dish at confluency, ∼1 × 10^7^ cells) expressing Myc-tagged fl-aPKCι WT or mutants were lysed in lysis buffer [50 mM Tris, pH 7.4, 150 mM NaCl, 1% Triton, 0.5 mM TCEP supplemented with phosphatase inhibitors (PhosSTOP, Roche) and protease inhibitors (cOmplete, Roche)] and immunoprecipitated using Myc-Trap agarose (Chromotek). Beads were washed once in lysis buffer containing 500 mM NaCl and twice in TBS. Equal amounts of the bead suspension were then added to 1.5 μg MBP and 250 μM ATP-γS in assay buffer (1× final; 50 mM Tris, 10 mM MgCl_2_). Reactions were incubated at 30 °C for 30 min and subsequently added with 2.5 mM p-Nitrobenzyl mesylate for 1.5 h at room temperature. Incorporation of S-labeled phosphates was followed *via* Western blotting with an anti-thiophosphate ester antibody.

### Differential scanning fluorometry

Protein at 250 μg/ml was incubated with SYPRO-orange protein dye (1:400) in 50 mM Tris, 150 mM NaCl, 0.5 mM TCEP with or without 1 mM ATP, and 10 mM MgCl_2_. The reactions were preincubated at room temperature for 5 min before being distributed into a MicroAmp 96-well qPCR plate. Samples were heated from 25 to 98 °C in a QuantStudio Flex 12 RT-PCR machine (ThermoFisher Scientific). Measurements of fluorescence emission at 570 nm were followed for each 0.3 °C increment. Raw curve data were exported, and derivative curves were plotted. The T_m_ of protein in each well was determined as the temperature at the inflection point of the melting curve using the maximal point of the derivative (51). The average melting temperature of at least three measurements was plotted, and protein conditions were compared *via* one-way ANOVA.

### Immunofluorescence

Cells (seeded at 0.1 × 10^6^/well of a 24-well plate) were grown on 13 mm glass coverslips, transfected with constructs as indicated, and 24 h post transfection, cultures were treated with 500 nM nocodazole for 16 h before fixation or left untreated. Cells were fixed with 4% PFA and permeabilized with PBS +0.1% Triton X-100. Coverslips were then blocked in 3% BSA in PBS and incubated with FLAG M2 antibody (1:500) and HA-Tag (C29F4) (1:1000) antibody where indicated. Secondary antibody was 1:1500 goat anti-mouse 488 (Thermo Fisher, A11001) or goat anti-rabbit 555 (Thermo Fisher Scientific, A21428). Coverslips were mounted using Prolong gold with DAPI (Thermo Fisher Scientific) and imaged. All the images were acquired using an inverted laser scanning confocal microscope (Carl Zeiss LSM 880) using a 63× Plan-APOCHROMAT DIC oil-immersion objective. The same exposure settings were used to acquire all images (excluding DAPI channel). Images shown in figures were processed in ZEN Blue edition (Zeiss) where a gold LUT pseudo-color transformation was applied to the 488-emission channel. All images were batch-processed to adjust brightness/contrast. Quantification of membrane and cytoplasmic intensities was done on the raw images in ImageJ. The whole membrane region was traced with the freehand tool, and the average pixel intensity was recorded. In the same way, the average intensity for a trace inside in a submembrane region of the cytoplasm was recorded, and the fraction of recorded intensities was plotted. For Src co-expression conditions, the average intensity of Src-HA signal was measured inside a circle corresponding to the recorded cell’s size and plotted against the ratio of membrane/cytosolic aPKC^RM^ protein quantified as above.

### Cell fractionation

To assess phosphorylation of Tyr-136 in the membrane and cytosolic fractions, HEK293T cells (10 cm dish at confluency, ∼1 × 10^7^ cells) expressing FLAG-tagged regulatory module were fractionated as in (52). Briefly, cells were lysed in homogenization buffer [20 mM Tris, pH 7.4, 10 mM EDTA, supplemented with phosphatase inhibitors (PhosSTOP, Roche), and protease inhibitors (cOmplete, Roche)] and subjected to sonication. The suspension was then centrifuged at 100,000*g* for 20′ at 4 °C, and the supernatant (Soluble fraction) was collected. The pellet (particulate fraction) was resuspended in homogenization buffer containing 1% Triton X-100, sonicated, and again centrifuged at 100,000*g* for 20′ at 4 °C. The supernatant was collected, and FLAG-IP was performed on both supernatants to isolate the regulatory domain for Western analysis with the pY136 antibody.

### Reduction-alkylation for immunodetection with pTyr-136 antibody

Western blots analysing levels of Tyr-136 phosphorylation were performed according to standard procedures but with the subsequent additional steps. After transfer, the nitrocellulose membrane was incubated overnight in PBS +10 mM DDT. It then was washed twice in PBS and incubated with PBS +10 mM iodoacetamide (Sigma) for 1 h at room temperature. The membrane was washed twice with PBS, and immunoblotting was continued according to standard procedures.

### Lipid overlay assay

aPKCι-Par6 WT and mutant complexes were purified as described above using 2xStrep-Par6 as bait. The protein complexes were analyzed *via* silver staining and incubated with lipid-coated membranes (PIP-Strips cat. P-6001) (Echelon Bioscience). The PIP strips were subsequently probed with Myc-antibody (1:1000) and HRP-tagged secondary antibody (1:3000). Membranes were processed and exposed in parallel in an ImageQuant LAS 4000 (GE HealthCare).

## Data availability

All the data described in this study are presented in the article and accompanying supporting information.

## Supporting information

This article contains supporting information (53).

## Conflict of interest

The authors declare that they have no conflicts of interest with the contents of this article.

## References

[1] Parker P.J., Brown S.J., Calleja V., Chakravarty P., Cobbaut M., Linch M. (2021). Equivocal, explicit and emergent actions of PKC isoforms in cancer. Nat. Rev. Cancer.

[2] Leroux A.E., Schulze J.O., Biondi R.M. (2018). AGC kinases, mechanisms of regulation and innovative drug development. Semin. Cancer Biol..

[3] Parker P.J., Justilien V., Riou P., Linch M., Fields A.P. (2014). Atypical protein kinase Cι as a human oncogene and therapeutic target. Biochem. Pharmacol..

[4] Graybill C., Wee B., Atwood S.X., Prehoda K.E. (2012). Partitioning-defective protein 6 (Par-6) activates atypical protein kinase C (aPKC) by pseudosubstrate displacement. J. Biol. Chem..

[5] Hirano Y., Yoshinaga S., Takeya R., Suzuki N.N., Horiuchi M., Kohjima M. (2005). Structure of a cell polarity regulator, a complex between atypical PKC and Par6 PB1 domains∗. J. Biol. Chem..

[6] Hirano Y., Yoshinaga S., Ogura K., Yokochi M., Noda Y., Sumimoto H. (2004). Solution structure of atypical protein kinase C PB1 domain and its mode of interaction with ZIP/p62 and MEK5∗. J. Biol. Chem..

[7] Buckley C.E., St Johnston D. (2022). Apical–basal polarity and the control of epithelial form and function. Nat. Rev. Mol. Cell Biol..

[8] Wodarz A., Ramrath A., Grimm A., Knust E. (2000). Drosophila atypical protein kinase C associates with bazooka and controls polarity of Epithelia and neuroblasts. J. Cell Biol..

[9] Franz A., Riechmann V. (2010). Stepwise polarisation of the Drosophila follicular epithelium. Dev. Biol..

[10] Wang S.-C., Low T.Y.F., Nishimura Y., Gole L., Yu W., Motegi F. (2017). Cortical forces and CDC-42 control clustering of PAR proteins for *Caenorhabditis elegans* embryonic polarization. Nat. Cell Biol..

[11] Martin-Belmonte F., Gassama A., Datta A., Yu W., Rescher U., Gerke V. (2007). PTEN-mediated apical segregation of phosphoinositides controls epithelial morphogenesis through Cdc42. Cell.

[12] Dong W., Lu J., Zhang X., Wu Y., Lettieri K., Hammond G.R. (2020). A polybasic domain in aPKC mediates Par6-dependent control of membrane targeting and kinase activity. J. Cell Biol..

[13] Jones K.A., Drummond M.L., Prehoda K.E. (2022). Cooperative regulation of C1-domain membrane recruitment polarizes atypical protein kinase C. bioRxiv.

[14] Mirdita M., Schütze K., Moriwaki Y., Heo L., Ovchinnikov S., Steinegger M. (2022). ColabFold: making protein folding accessible to all. Nat. Methods.

[15] Kufareva I., Lenoir M., Dancea F., Sridhar P., Raush E., Bissig C. (2014). Discovery of novel membrane binding structures and functions. Biochem. Cell Biol..

[16] Hornbeck P.V., Zhang B., Murray B., Kornhauser J.M., Latham V., Skrzypek E. (2015). PhosphoSitePlus, 2014: mutations, PTMs and recalibrations. Nucleic Acids Res..

[17] Schaab C., Geiger T., Stoehr G., Cox J., Mann M. (2012). Analysis of high accuracy, quantitative proteomics data in the MaxQB database. Mol. Cell. Proteomics.

[18] Cobbaut M., Derua R., Parker P.J., Waelkens E., Janssens V., Van Lint J. (2018). Protein kinase D displays intrinsic Tyr autophosphorylation activity: insights into mechanism and regulation. FEBS Lett.

[19] Blom N., Gammeltoft S., Brunak S. (1999). Sequence and structure-based prediction of eukaryotic protein phosphorylation sites. J. Mol. Biol..

[20] Miller M.L., Jensen L.J., Diella F., Jørgensen C., Tinti M., Li L. (2008). Linear motif atlas for phosphorylation-dependent signaling. Sci. Signal..

[21] Smolinski M.P., Bu Y., Clements J., Gelman I.H., Hegab T., Cutler D.L. (2018). Discovery of novel dual mechanism of action src signaling and tubulin polymerization inhibitors (KX2-391 and KX2-361). J. Med. Chem..

[22] Kajimoto T., Caliman A.D., Tobias I.S., Okada T., Pilo C.A., Van A.-A.N. (2019). Activation of atypical protein kinase C by sphingosine 1-phosphate revealed by an aPKC-specific activity reporter. Sci. Signal..

[23] Elbediwy A., Zhang Y., Cobbaut M., Riou P., Tan R.S., Roberts S.K. (2019). The Rho family GEF FARP2 is activated by aPKCι to control tight junction formation and polarity. J. Cell Sci..

[24] Golub O., Wee B., Newman R.A., Paterson N.M., Prehoda K.E. (2017). Activation of discs large by aPKC aligns the mitotic spindle to the polarity axis during asymmetric cell division. Elife.

[25] Soriano E.V., Ivanova M.E., Fletcher G., Riou P., Knowles P.P., Barnouin K. (2016). aPKC inhibition by Par3 CR3 flanking regions controls substrate access and underpins apical-junctional polarization. Dev. Cell.

[26] Yamanaka T., Horikoshi Y., Sugiyama Y., Ishiyama C., Suzuki A., Hirose T. (2003). Mammalian Lgl forms a protein complex with PAR-6 and aPKC independently of PAR-3 to regulate epithelial cell polarity. Curr. Biol..

[27] Sotillos S., Díaz-Meco M.T., Caminero E., Moscat J., Campuzano S. (2004). DaPKC-dependent phosphorylation of crumbs is required for epithelial cell polarity in Drosophila. J. Cell Biol..

[28] Hurov J.B., Watkins J.L., Piwnica-Worms H. (2004). Atypical PKC phosphorylates PAR-1 kinases to regulate localization and activity. Curr. Biol..

[29] Gamblin C.L., Parent-Prévost F., Jacquet K., Biehler C., Jetté A., Laprise P. (2018). Oligomerization of the FERM-FA protein yurt controls epithelial cell polarity. J. Cell Biol..

[30] Shahab J., Tiwari M.D., Honemann-Capito M., Krahn M.P., Wodarz A. (2015). Bazooka/PAR3 is dispensable for polarity in Drosophila follicular epithelial cells. Biol. Open.

[31] Kanemaru K., Shimozawa M., Kitamata M., Furuishi R., Kayano H., Sukawa Y. (2022). Plasma membrane phosphatidylinositol (4,5)-bisphosphate is critical for determination of epithelial characteristics. Nat. Commun..

[32] Lu J., Dong W., Hammond G.R., Hong Y. (2022). Hypoxia controls plasma membrane targeting of polarity proteins by dynamic turnover of PI4P and PI(4,5)P2. Elife.

[33] Bailey M.J., Prehoda K.E. (2015). Establishment of par-polarized cortical domains via phosphoregulated membrane motifs. Dev. Cell.

[34] Brzeska H., Guag J., Remmert K., Chacko S., Korn E.D. (2010). An Experimentally based computer search identifies unstructured membrane-binding sites in proteins. J. Biol. Chem..

[35] Mosior M., McLaughlin S. (1991). Peptides that mimic the pseudosubstrate region of protein kinase C bind to acidic lipids in membranes. Biophys. J..

[36] Ivey R.A., Sajan M.P., Farese R.V. (2014). Requirements for pseudosubstrate arginine residues during autoinhibition and phosphatidylinositol 3,4,5-(PO4)3-dependent activation of atypical PKC. J. Biol. Chem..

[37] Acs P., Beheshti M., Szállási Z., Li L., Yuspa S.H., Blumberg P.M. (2000). Effect of a tyrosine 155 to phenylalanine mutation of protein kinase cdelta on the proliferative and tumorigenic properties of NIH 3T3 fibroblasts. Carcinogenesis.

[38] Humphries M.J., Ohm A.M., Schaack J., Adwan T.S., Reyland M.E. (2008). Tyrosine phosphorylation regulates nuclear translocation of PKCδ. Oncogene.

[39] Kronfeld I., Kazimirsky G., Lorenzo P.S., Garfield S.H., Blumberg P.M., Brodie C. (2000). Phosphorylation of protein kinase cδ on distinct tyrosine residues regulates specific cellular functions∗. J. Biol. Chem..

[40] Steinberg S.F. (2004). Distinctive activation mechanisms and functions for protein kinase Cδ. Biochem. J..

[41] Meyer T.N., Schwesinger C., Ye J., Denker B.M., Nigam S.K. (2001). Reassembly of the tight junction after oxidative stress depends on tyrosine kinase activity. J. Biol. Chem..

[42] Tsukamoto T., Nigam S.K. (1999). Role of tyrosine phosphorylation in the reassembly of occludin and other tight junction proteins. Am. J. Physiol..

[43] Yap A.S., Stevenson B.R., Cooper V., Manley S.W. (1997). Protein tyrosine phosphorylation influences adhesive junction assembly and follicular organization of cultured thyroid epithelial cells. Endocrinology.

[44] Wooten M.W., Vandenplas M.L., Seibenhener M.L., Geetha T., Diaz-Meco M.T. (2001). Nerve growth factor stimulates multisite tyrosine phosphorylation and activation of the atypical protein kinase C’s via a src kinase pathway. Mol. Cell. Biol..

[45] Suzuki A., Akimoto K., Ohno S. (2003). Protein kinase C λ/ι (PKCλ/ι): a PKC isotype essential for the development of multicellular organisms. J. Biochem..

[46] Anton K.A., Kajita M., Narumi R., Fujita Y., Tada M. (2018). Src-transformed cells hijack mitosis to extrude from the epithelium. Nat. Commun..

[47] Okuyama H., Kondo J., Sato Y., Endo H., Nakajima A., Piulats J.M. (2016). Dynamic change of polarity in primary cultured spheroids of human colorectal adenocarcinoma and its role in metastasis. Am. J. Pathol..

[48] Morinaga T., Yanase S., Okamoto A., Yamaguchi N., Yamaguchi N. (2017). Recruitment of Lyn from endomembranes to the plasma membrane through calcium-dependent cell-cell interactions upon polarization of inducible Lyn-expressing MDCK cells. Sci. Rep..

[49] Luo J., Mcginnis L.K., Kinsey W.H. (2009). Fyn kinase activity is required for normal organization and functional polarity of the mouse oocyte cortex. Mol. Reprod. Dev..

[50] Ashkenazy H., Abadi S., Martz E., Chay O., Mayrose I., Pupko T. (2016). ConSurf 2016: an improved methodology to estimate and visualize evolutionary conservation in macromolecules. Nucleic Acids Res..

[51] Niesen F.H., Berglund H., Vedadi M. (2007). The use of differential scanning fluorimetry to detect ligand interactions that promote protein stability. Nat. Protoc..

[52] Wescott G.G., Manring C.M., Terrian D.M. (1999). Translocation assays of protein kinase C activation. Methods Mol. Med..

[53] Shah N.H., Löbel M., Weiss A., Kuriyan J. (2018). Fine-tuning of substrate preferences of the Src-family kinase Lck revealed through a high-throughput specificity screen. Elife.

