# Supplementary material for: Control of atypical PKCι membrane dissociation by tyrosine phosphorylation within a PB1-C1 interdomain interface
Source: J Biol Chem. 2023 May 20;299(7):104847. doi: 10.1016/j.jbc.2023.104847 (PMC10333572; doi:10.1016/j.jbc.2023.104847)
Supplement: Supporting Figures S1–S3 and Tables S1–S4 [file mmc1.docx]

# Supporting information for

# Control of atypical PKCι membrane dissociation by tyrosine phosphorylation within a PB1-C1 interdomain interface

Mathias Cobbaut^1,2*^, Neil Q. McDonald^1,3*^, Peter J. Parker^2.4*^

^1^ Signalling and Structural Biology Laboratory, The Francis Crick Institute, 1 Midland Road, London, NW1 1AT, UK

^2^ Protein Phosphorylation Laboratory, The Francis Crick Institute, 1 Midland Road, London, NW1 1AT, UK

^3^ Institute of Structural and Molecular Biology, Department of Biological Sciences, Birkbeck College, London, WC1E 7HX, UK.

^4^ School of Cancer and Pharmaceutical Sciences, King's College London, Guy's Campus, London, SE1 1UL, UK.

* for correspondence: Mathias Cobbaut, [mathias.cobbaut@crick.ac.uk](mailto:mathias.cobbaut@crick.ac.uk); Neil Q. McDonald, [neil.mcdonald@crick.ac.uk](mailto:neil.mcdonald@crick.ac.uk); Peter J. Parker, [peter.parker@crick.ac.uk](mailto:peter.parker@crick.ac.uk)

Contents

[Figure S1 2](#_Toc124513746)

[Figure S2 3](#_Toc124513747)

[Figure S3 4](#_Toc124513748)

[Table S1 5](#_Toc124513749)

[Table S2 7](#_Toc124513750)

[Table S3 9](#_Toc124513751)

[Table S4 9](#_Toc124513752)

## Figure S1


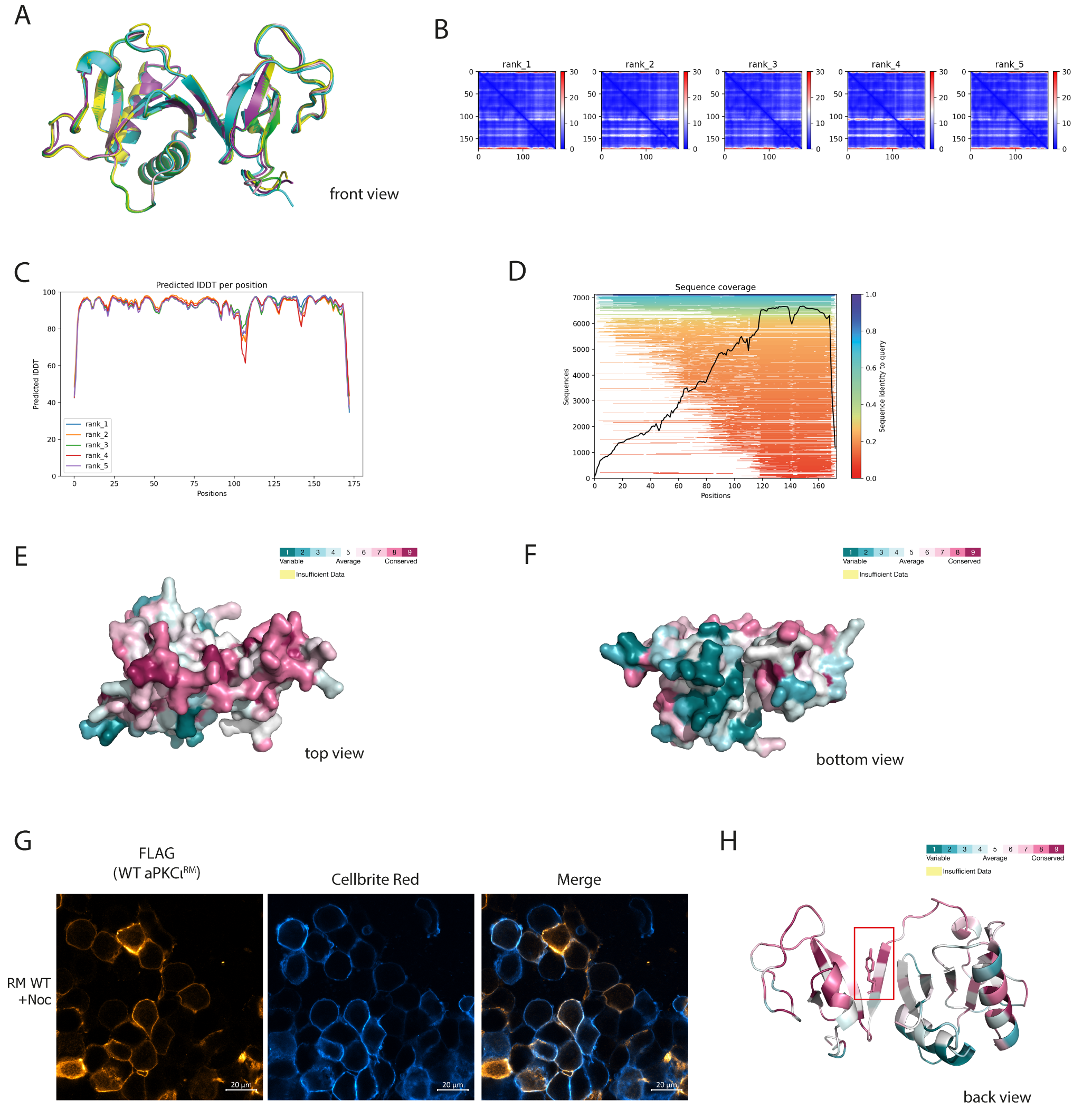


Fig S1. A. Overlay of the five top scoring Alphafold Colab models; Rank1 (green), Rank 2 (cyan), Rank 3 (purple), Rank 4 (yellow), Rank 5 (salmon). B. predicted alignment error (PAE) for the different models. C. plDDT per residue for the different models. D. sequence coverage of the multiple sequence alignment. E. Top view of the regulatory module coloured by conservation as analysed by ConSurf (Red: high conservation – Green low conservation). F. Bottom view of the aPKCι regulatory module coloured by conservation as analyzed by ConSurf. G. Staining of HEK293T cells arrested with Nocodazole with FLAG-M2 antibody and the membrane marker Cellbrite Red. Cells were mounted in PBS. H. Cartoon representation of the aPKCι regulatory module indicating the conserved Tyr residue central to the BSL.

## Figure S2


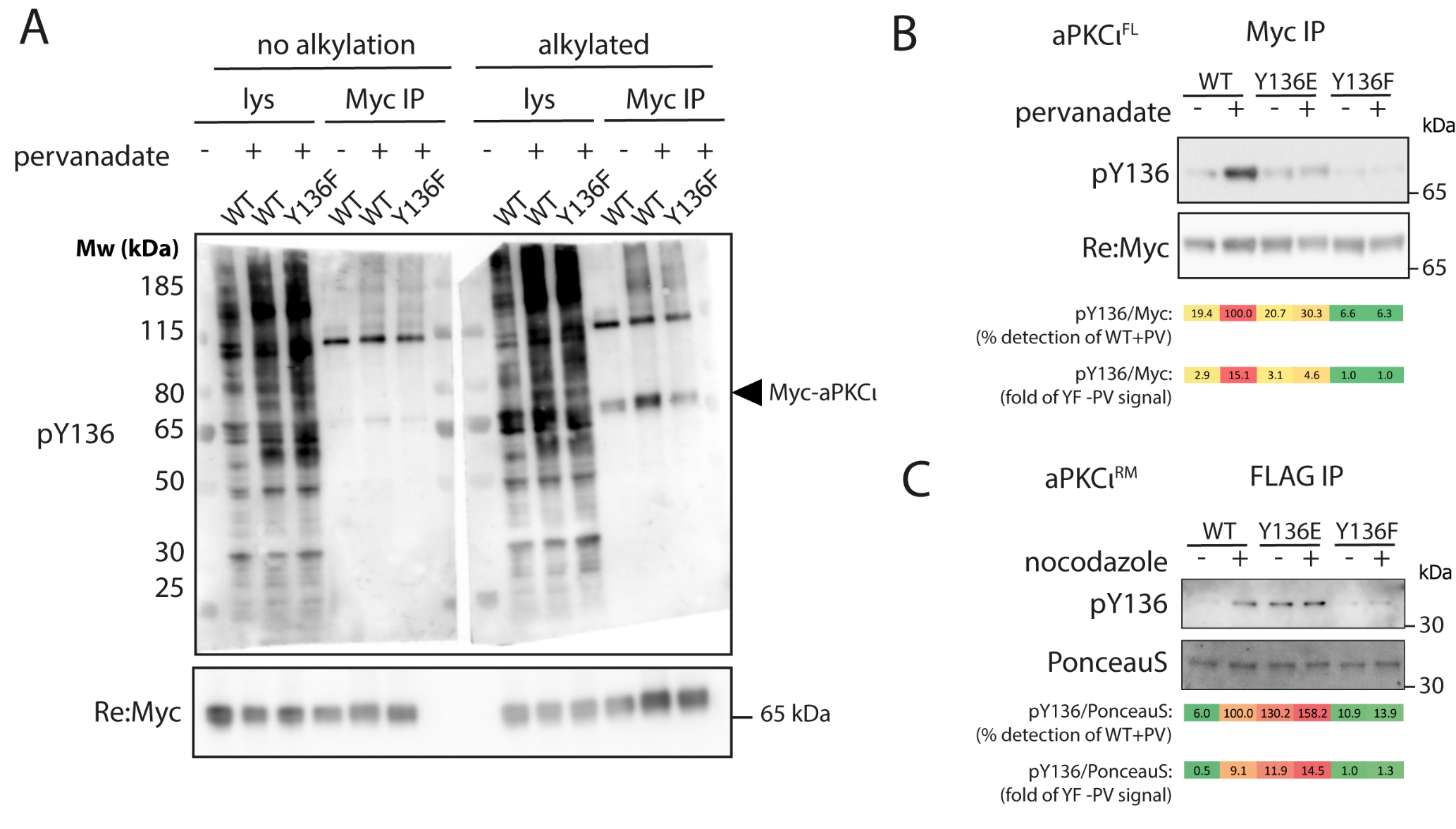


Fig S2. Specificity of the phosho-Tyr136 antibody. A. cells transfected with the indicated constructs were treated with pervanadate or left untreated. Lysates and Myc immunoprecipitates were loaded on an SDS-PAGE gel, transferred to nitrocellulose and subjected to a reduction-alkylation step or left untreated; and subsequently analysed with the pTyr-136 antibody. The antibody shows low specificity in cell lysates with broad recognition of pervanadate responsive signals, but the immunoprecipitated kinase is specifically recognized after pervanadate stimulation only when Tyr-136 is intact and post-alkylation. B. Validation of the specificity of the antibody towards immunoprecipitated Myc-aPKCι and mutants with prior alkylation. C. Validation of the specificity of the antibody towards immunoprecipitated aPKCι^RM^ and mutants with prior alkylation.

## Figure S3


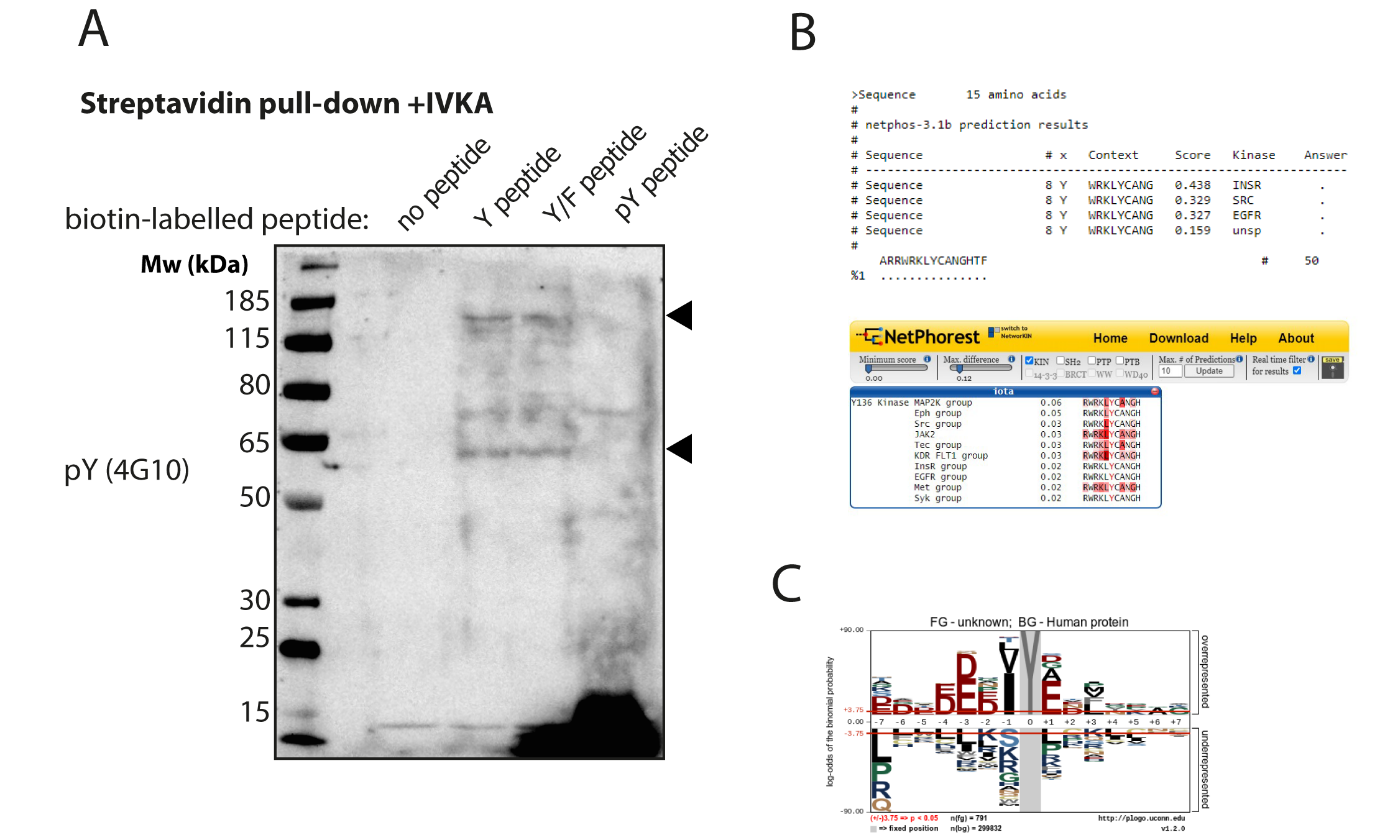


Fig S3. A. Tyr phosphorylation activity detection after precipitation of proteins from cell extracts with the indicated pseudo-substrate peptides. The precipitates were spiked with ATP.Mg^2+^ for 60’ and tyrosine (auto)phosphorylation was followed with the pY (4G10) antibody. Bands were detected for non-phosphorylated Y and Y/F peptide around ~60 and ~170 kDa (arrowheads). B. Upstream kinase predictions for Tyr-136 in Netphos3.1 and NetPhorest. C. Kinase recognition motif for Src analysed using data from Shah et al. 2018. All substrates with an enrichment score >3 for c-Src were analysed for enrichment v.a.v. the human proteome background using pLogo.

## Table S1

MODA per-residue prediction scores


Table S2

## Table S3

phosphorylation sites identified for aPKCι in the MaxQuant database (MaxQB)

| **Sequence** | **Type** | **Residue** | **Position** | **Identifications** | **Best Score** | **Best Score Diff** | **Best Localization Probability** | **Regions** | **Conserved In** |
| --- | --- | --- | --- | --- | --- | --- | --- | --- | --- |
| PGDTTST(ph)FCGTPNY | Phospho (STY) | T | 11 | 2 | 121.23 | 6.63664 | 0.808082 | Required for interaction with RAB2, Regulatory domain |  |
| TVAGGGS(ph)GDHSHQV | Phospho (STY) | S | 19 | 1 | 205.12 | 25.6809 | 0.997304 | Required for interaction with RAB2, Regulatory domain |  |
| RRWRKLY(ph)CANGHTF | Phospho (STY) | Y | 136 | 1 | 57.175 | 43.9897 | 0.99996 | Regulatory domain |  |
| NEPVQLT(ph)PDDDDIV | Phospho (STY) | T | 564 | 1 | 364.21 | 108.536 | 1 | AGC-kinase C-terminal |  |

## Table S4

Primers and constructs used in this study
